# Common B-cell acute lymphoblastic leukaemia in a 70-year-old woman presenting 2 years after carboplatin-taxane radiotherapy for endometrial cancer

**DOI:** 10.3332/ecancer.2019.972

**Published:** 2019-10-29

**Authors:** Nigel P Murray, Shenda Orrego, Marco Antonio López, Lorena Munoz, Simona Minzer

**Affiliations:** 1 Consultant Haematologist, Department of Medicine, Hospital de Carabineros de Chile, Simón Bolívar 2200, Ñuñoa, Santiago, 7770199, Chile; 2 Professor Haematology, Faculty of Medicine, University Finis Terrae, Av Pedro de Valdivia 1509, Providencia, Santiago; 3 Physician General Medicine, Department of Medicine, Hospital de Carabineros de Chile, Simón Bolívar 2200, Ñuñoa, Santiago, 7770199, Chile; 4 Tutor, Faculty of Medicine, University Mayor, Renato Sánchez 4369, Las Condes, Santiago, 27550224, Chile; 5 Consultant Internal Medicine, Department of Medicine, Hospital de Carabineros de Chile, Simón Bolívar 2200, Ñuñoa, Santiago, 7770199, Chile

**Keywords:** therapy-related acute lymphoblastic leukaemia, endometrial cancer, carboplatin, paclitaxel

## Abstract

Therapy-related acute lymphoblastic leukaemia (t-ALL) is a poorly defined entity and is not featured in the World Health Organization classification as a distinct clinical entity from acute lymphoblastic leukaemia (ALL), thus differing from therapy-related acute myeloid leukaemia and myelodysplasia. We present a case of t-ALL occurring 18 months after treatment for metastatic endometrial cancer with a regimen of carboplatin, paclitaxel and radiotherapy. The patient presented with severe pancytopenia and diagnosed with common-B ALL, and the cytogenetic analysis showed a previously unreported deletion in chromosome 19 (q13.1) in 100% of the blast cells. The patient declined further therapy and died 1 month later. This rare but serious side effect of chemo-radiotherapy should be considered when deciding on treatment options for gynaecological cancers.

## Introduction

Therapy-related leukaemia is a long-term side effect of chemotherapy and/or radiotherapy in patients who have been treated for a previous cancer. Therapy-related myelodysplasia and acute myeloid leukaemia (AML) are an established category of the World Health Organization classification of myeloid neoplasms [1]. However, therapy-related acute lymphoblastic leukaemia (t-ALL) is a poorly defined entity and typically is not considered as a treatment-related complication due to a lack of large data sets that recognise the defining characteristics of this cancer [2]. Only a few relatively small series have been reported [3–9] and have included cases with a previous history of cancer but without cytotoxic or radiation exposure. Additional limitations of these studies have included a lack of cytogenetic analysis and specific details of previous treatments. We have used the definition that restricts t-ALL to those patients treated with chemotherapy and/or radiotherapy.

t-ALL is infrequent representing 2%–9% of all acute lymphoblastic leukaemia (ALL) cases [3–5] and 10%–15% of all therapy-related leukaemia’s [3–5]. Rosenberg *et al* [4] analysed 14,470 patients with ALL using the California Cancer Registry data from 1988 to 2016; of this population of ALL patients, 3% of cases had been treated for a previous malignancy. A more recent analysis of 4,851,222 first cancer patients reported that 849 (0.02%) patients developed ALL as a second cancer. In comparison with the general population, the relative risk of developing ALL in the first 10 years after completing treatment was 1.59 for patients treated with only radiotherapy; 3.47 for those treated with chemotherapy and 3.22 for those treated with combined therapy. Patients’ not receiving chemotherapy or radiotherapy for their first cancer had no increased risk for ALL [2].

We present the case of a 70-year-old woman who presented with a pancytopenia 2 years after receiving chemo-radiotherapy for endometrial cancer. We discuss the treatment options and cytogenetic findings.

## Clinical case

A 70-year-old woman presented to the emergency service with a 1-month history of progressive dyspnoea and tachycardia with minimal activity. A full blood count revealed a haemoglobin level of 4.3 gr/dL, platelet count 7,000/mm^3^ and a white cell count of 7,200/mm^3^ with an absolute neutrophil count of 360/mm^3^. The total serum lactate dehydrogenase was 427 U/L (normal range 0–223), serum uric acid was 7.0 mg/dL (normal range 2.3–6.1) and a serum total alkaline phosphatase of 111 U/L (normal range 0–105).

Analysis of the blood smear showed small- and medium-sized lymphocytes, some with a nucleolus.

Three years earlier, the patient had presented to the gynaecologist with pelvic pain without haemorrhage. A pelvic ultrasound revealed a uterine tumour and an endometrial biopsy confirmed an endometrial adenocarcinoma grade 2 with a focus of serous carcinoma. Immunohistochemistry showed positivity for vimentin, 80% of tumour cells were positive for the oestrogen receptor and negative for the expression of carcinoembryonic antigen. P53 expression was weakly positive in the adenocarcinoma component and strongly positive in 90% of the serous carcinoma component.

The patient was operated in September 2015, undergoing a total hysterectomy, bilateral salpingo-oophorectomy, omentectomy, pelvic lymphadenectomy and samples for peritoneal cytology were taken. An intra-operative biopsy showed that the left obturator lymph node was 1.5 cm in diameter and infiltrated with adenocarcinoma. The uterus weighed 162 g with the uterine cavity occupied by tumour that was haemorrhagic in nature. It partially infiltrated the myometrium and extended distally to occupy the upper third of the cervix. Both ovaries and fallopian tubes were free of tumour. The omentum was free of tumour and peritoneal cytology was negative for tumour cells. The cancer comprised an exo-phytic growth of 4 cm × 2 cm × 2.5 cm with infiltration of the lymphovascular structures and up to 50% of the myometrium. There was a superficial invasion of the upper third of the cervix and metastasis in the left obturator and left hypogastric lymph nodes. In 17 other lymph nodes examined, there was no evidence of metastasis.

The patient’s recovery was complicated by a pulmonary embolus and was treated for 6 months with rivaroxaban.

After surgery, the patient underwent six cycles of carboplatin-paclitaxel chemotherapy, which was completed in February 2016 and without dose reduction. In May 2016, she underwent two treatments of brachytherapy of 11 Gy to the vaginal vault and in June 2016 treated with external beam six-field conformational radiotherapy, with a dose of 45 Gy in 25 fractions. There was no evidence of metastatic spread on imaging studies at this time.

However, in October 2016, a routine CT scan of the lungs showed a solid nodule irregular in the outline of 28 mm in the right inferior lobe, one of 18 mm in the right superior lobe and one of 15 mm in the left superior lobe of the lung. There was no evidence of relapse in the abdomen or pelvis. She underwent eight further cycles of carboplatin-paclitaxel, finishing treatment in March 2017.

The patient was hospitalised in December 2018, with a severe pancytopenia and the presence of blasts in the blood smear. Repeat CT scan did not show any pulmonary nodules or evidence of metastatic disease in the abdomen or pelvis.

She was treated initially with transfusions of filtered packed red cells and platelets, and a working diagnosis of acute secondary leukaemia with or without a previous phase of myelodysplasia.

**Figure 1.**
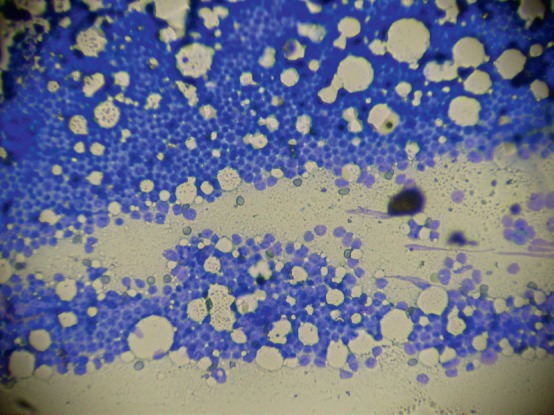
Low power magnification ‘touch preparation’ bone marrow biopsy showing dense infiltration with lymphoid type blasts.

**Figure 2.**
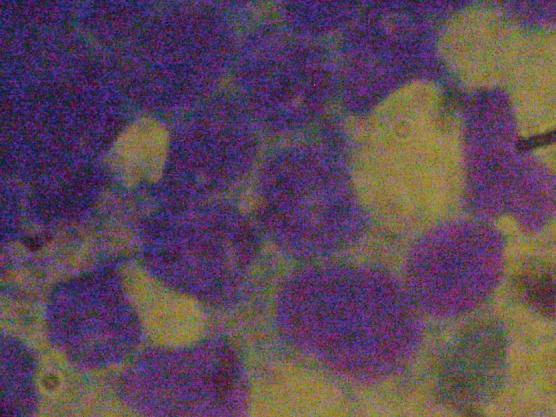
Magnification × 400 showing blasts with an undifferentiated appearance and some with vacuoles.

Bone marrow aspiration of the right posterior superior iliac crest resulted in a ‘dry tap’ and a bone marrow biopsy showed an infiltration of 95% blasts morphologically lymphoid in nature (Figures 1 and 2). A sternal bone marrow aspirate was obtained for immunophenotyping and cytogenetic studies.

Flow cytometry showed an infiltration of 98% blasts, which were positive for the B-cell markers CD19 (99% positive), human leukocyte antigen-DR (HLA-DR) (99%), CD10 (53%) and negative for CD20. The blasts were negative for the T-cell markers CD2, CD3 and CD7 and for the myeloid markers CD14, CD15, CD33, CD11c, CD64 and CD117.

79% of the blasts were positive for cytoplasmic CD79a and 51% for CD34 and were negative for cytoplasmic CD3, myeloperoxidase, IgM and lysozyme.

**Figure 3.**
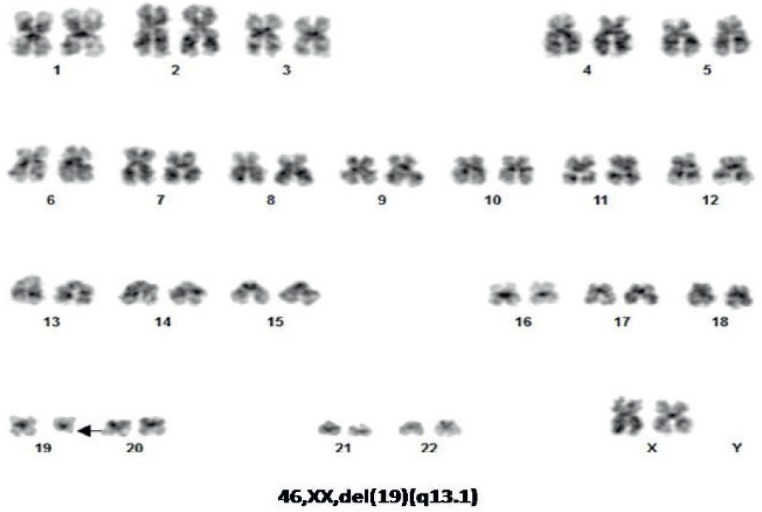
Karyotype analysis showing del (19) (q13.1).

Using the reverse transcriptase polymerase chain reaction, the blasts were negative for the t [9, 21] p190 and p210 variants of the BCR-ABL1 fusion genes and for the t [4, 10] fusion gene MLL-AF4. Cytogenetic analysis of G-banded chromosomes showed a karyotype 46, XX, deletion chromosome 19 (q13.1) in 100% of the blast cells (Figure 3).

A diagnosis of treatment-related acute B-cell common type lymphoblastic leukaemia was made. The patient declined chemotherapy, was treated with transfusions as required and died 1 month later.

## Discussion

t-ALL has been divided into two major types; firstly, in those patients who had received alkylating agents and/or radiotherapy, the mean latency period between treatment and the presentation of acute leukaemia is between 5 and 7 years. The most commonly used alkylating agents being cyclophosphamide, melphalan and nitrosourea. The platinum containing derivatives, cisplatin and carboplatin are carcinogenic *in vitro* and in laboratory animals producing intra-strand and inter-strand DNA cross links in a manner similar to that of bi-functional alkylating agents. The relative risk of developing leukaemia after receiving a median dose of 3,300 mg of carboplatin has been reported as 6.5 times that of age-matched control patients [10]. The combined use of radiotherapy with carboplatin increased this relative risk to 8.1 [10]. Carboplatin has been reported to be associated with treatment-related myelodysplasia and AML [10]. These treatment-related leukaemias are associated with complex karyotype abnormalities and monosomy 5 or 7 [10, 11]. Monosomy or deletion of the long arm of chromosomes 5 and 7 is more common in t-ALL than *de novo* ALL, 16% versus 8%, respectively [4]. Radiation to the pelvis, which affects approximately 50% of the active bone marrow in adults, is associated with a three-fold increase in leukaemia [12]. It has been reported that for every 10,000 patients treated with radiotherapy at a dose of 25Gy for testicular cancer, an excess of nine leukaemia cases will be seen after 15 years of follow-up. As such the number of cases of t-ALL following radiotherapy is small in comparison with the potential benefits [12]. This has to be put in context with the survival advantage of treatment. Similarly, in patients treated with radiotherapy for cancer of the cervix, an increased risk of secondary leukaemia has been reported [13].

The second type of t-ALL is seen in patients treated with DNA topoisomerase II inhibitors; in these patients, there is a short latency period of 1–3 years between cancer treatment and development of t-ALL, typically in these patients, a myelodysplastic phase is not detected. In this type of t-ALL, there is an association with a chromosomal translocation involving the 11q23, mixed-lineage leukaemia (MLL) gene locus, although cases without this translocation have been described [14, 15]. MLL rearrangements have been described for both AML and ALL representing approximately 5% of therapy-related acute leukaemia. This rearrangement is more frequently found in t-ALL compared to t-AML and is associated with CD20 negative pro-B-cell acute lymphoblastic leukaemia (ALL) [16, 17]. Differing from treatment-related AML, in t-ALL, the 11q23 translocation appears to occur in patients treated with or without DNA topoisomerase II inhibitors [18] and is more frequently detected in t-ALL than in *de novo* ALL (17% versus 4%, respectively) [4]. With respect to prior treatment with taxanes, case reports of both paclitaxel [19] and docetaxel [20] have been associated with 11q23 t-AML.

The most common chromosomal abnormality reported in t-ALL is the 11q23 MLL translocation; followed by the BCL-ABL translocation. The presence of the BCL-ABL translocation in t-ALL has been associated with previous radiotherapy [16, 18]. The third commonest karyotype seen in t-ALL is a normal karyotype. However, it must be remembered that obtaining good quality metaphase cells in ALL is often suboptimal and cryptic aberrations may not be identified in all patient samples [14].

The chromosomal abnormality del (19) (q13.1) as the only cytogenetic abnormality has not been previously reported in t-ALL. Abnormalities involving chromosome 19 in patients with t-ALL have been previously reported; t (1:19) (q23: p13.1) involving the MLL1/ELL genes, t (1:19) (q23: p13.1) involving the MLL1/ENL genes, t (1:19) (q23:p13) involving the E2A/PBX genes and t (17:19) (q22:p13) involving the E2A/HLF genes [21]. The French Cytogenetics in Haematology Group in a study of 443 karyotypes did not report the del (19) (q13.1) as an abnormal karyotype found in ALL [22]. A pooled analysis of cytogenetic features in treatment-related myelodysplasia did not report the del (19) (q13.1) abnormality [23]. In a further report of 1,522 patients and cytogenetic analysis, the del (19) (q 13.1) was not reported [24], while one case of monosomy 19 was reported in 881 cases [25].

Why some patients develop t-ALL years after receiving radio-chemotherapy and others do not is unknown. *In vitro* studies to determine the chromosomal instability have used peripheral blood lymphocytes as a marker for the dysfunction of DNA reparation genes have suggested that patients with increased chromosomal instability have an increased risk of second cancers when exposed to chemotherapy or radiotherapy [26]. Thus, it may be that patients with higher chromosomal instability have a higher risk of developing t-ALL.

Treatment options depend on patient age and co-morbidities, with older patients having a worse outcome. Where possible, a bone marrow transplant is recommended for younger patients with suitable donors. It is associated with a lower rate of relapse but there is increased transplant-related mortality with no overall increase in survival [27]. In those not eligible for transplant, the outcome to standard treatment is poorer than in *de novo* ALL [28]. There are no large-scale trails of t-ALL treatment; various chemotherapy protocols have been used, commonly with reduced dose in the more elderly patients and as such, there is no consensus on treatment. Relapse rates after standard treatment are relatively high and survival is low, which is likely related to the high frequency of adverse cytogenetic abnormalities.

In those patients positive for BCL-ABL1, the use of tyrosine kinase inhibitors has been used in combination with chemotherapy, although with the small number of cases, the effect of prognosis is not known [29, 30].

In summary with the increased use of combined chemo-radiotherapy in patients with gynaecological or urological cancers, as adjuvant therapy or for the treatment of metastasis, the number of patients presenting with t-ALL may increase. We presented a case of treatment-related acute B-cell lymphoblastic leukaemia with a previously unreported chromosomal abnormality. Although the patient did not have evidence of metastasis 2 years after combined chemo-radiotherapy, the patient decided against treatment for the t-ALL. Oncologists should be aware of this rare but significantly severe adverse effect and it should be kept in consideration when deciding on treatment options for these patients, especially concerning patient age and co-morbidities. Due to the paucity of cases and lack of consensus on treatment, multicentre studies or registers are warranted to accumulate evidence of treatment options and outcomes.

## Conflicts of interest

The authors declare that they have no conflicts of interest.

## Funding declaration

No financial support was received for this case report.

## Ethical approval

The case study was approved by the local ethics committee and in complete conformity with the Declaration of Helsinki and the Chilean law on patient’s rights.

## References

[1] Arber DA, Orazi A, Hasserjian R (2016). The 2016 revision to the World Health Organization classifications of myeloid neoplasms and acute leukemia. Blood.

[2] Molenaar RJ, Radivoyevitch T, Gerds AT (2018). Is there an increased risk of ALL in patients with first cancers treated with radiotherapy and/or chemotherapy?. Blood.

[3] Aldoss I, Stiller T, Tsai NC (2018). Therapy related acute lymphoblastic leukemia has distinct clinical and cytogenetic features compared with de novo acute lymphoblastic leukemia, but outcomes are comparable in transplanted patients. Haematologica.

[4] Rosenberg AS, Brunson A, Paulus JK (2017). Secondary acute lymphoblastic leukemia is a distinct clinical entity with prognostic significance. Blood Cancer J.

[5] Swaika A, Frank RD, Yang D (2018). Secondary primary acute lymphoblastic leukemia in adults: a SEER analysis of incidence and outcomes. Cancer Medicine.

[6] Toft N, Schmieglow K, Klausen TW (2012). Adult ALL in Denmark. A national population based retrospective study on ALL in Denmark 1998–2008. Br J Haematol.

[7] Kelleher N, Gallardo D, Gonzalez-Campos J (2016). Incidence, clinical and biological characteristics and outcome of secondary acute lymphoblastic leukemia after solid organ or hematologic malignancy. Leuk Lymphoma.

[8] Shivakumar R, Tan W, Wilding GE (2008). Biological features and treatment outcome of secondary acute lymphoblastic leukemia-a review of 101 cases. Annals Oncol.

[9] Pagano L, Pulsoni A, Tosti ME (2001). Clinical and biological features of acute myeloid leukaemia occurring as second malignancy: GIMEMA archive of adult acute leukaemia. Br J Haematol.

[10] Travis LB, Holowaty EJ, Bergfeldt K (1999). Risk of leukemia after platinum-based chemotherapy for ovarian cancer. N Eng J Med.

[11] Smith SM, LeBeau MM, Huo D (2003). Clinical-cytogenetic associations in 306 patients with therapy related myelodysplasia and myeloid leukemia: the University of Chicago series. Blood.

[12] Travis LB, Andersson M, Gospodarowicz M (2000). Treatment associated leukemia following testicular cancer. J Natl Cancer Inst.

[13] Boice JD, Biettner M, Kleinerman RA (1987). Radiation dose and leukemia risk in patients treated for cancer of the cérvix. J Natl Cancer Inst.

[14] Shivakumar R, Tan W, Wilding GE (2008). Biologic features and treatment outcome of secondary acute lymphoblastic leukemia-a review of 101 cases. Annals Oncol.

[15] Chen W, Wang E, Lu Y (2010). Therapy related acute lymphoblastic leukemia without 11q23 abnormality. Am J Clin Pathol.

[16] Pagano L, Pulsoni A, Mele L (2000). Clinical and epidemiological features of acute lymphoblastic leukemia following a previous malignancy. Leuk Lymphoma.

[17] Ishizawa S, Slovak ML, Popplewell L (2003). High frequency of pro-B acute lymphoblastic leukemia in adults with secondary leukemia with 11q23 abnormalities. Leukemia.

[18] Abdulwahab A, Sykes J, Kamel-Reid S (2012). Therapy related acute lymphoblastic leukemia is more frequent than previously recognized and has a poor prognosis. Cancer.

[19] Saito M, Mori A, Irie T (2009). Therapy related acute myeloid leukemia with 11q23 abnormality due to paclitaxel coexisting with bone marrow metástasis of breast cancer. Rinsho Ketsueki.

[20] Numakura K, Tsichiya N, Habuchi T (2009). Therapy related leukemia with 11q23 abnormality induced by chemotherapy consisted of docetaxel for advanced prostatic carcinoma: case report. Nihon Hinyokika Gakkai Zasshi.

[21] Choi HJ, Kim HR, Shin MG (2011). Spectra of chromosomal aberrations in 325 leukemia patients and implications for the development of new molecular detection systems. J Korean Med Sci.

[22] Groupe Francais de Cytogenetique Hematologique (1996). Cytogenetic abnormalities in adult acute lymphoblastic leukemia: correlations with hematologic findings and outcome. A collaborative study of the Group Francais de Cytogenetique Hematolgique. Blood.

[23] Mauritzson N, Albin M, Rylander L (2002). Pooled analysis of clinical and cytogenetic features in treatment related and de novo adult acute myeloid leukemia and myelodysplastic syndromes based on a consecutive series of 761 patients analyzed 1976-1993 and on 5098 unselected cases reported in the literature 1974-2001. Leukemia.

[24] Moorman AV, Harrison CJ, Buck GAN (2007). Karyotype is an independent prognostic factor in adult acute lymphoblastic leukemia (ALL): analysis of cytogenetic data from patients treated on the Medical Research Council (MRC) UKALLXII/Eastern Cooperative Oncology Group (ECOG) 2993 trail. Blood.

[25] Motilo C, Ribera JM, Morgades M (2014). Prognostic significance of complex karyotype and monosomal karyotype in adult patients with acute lymphoblastic leukemia treated with risk adapted protocols. Cancer.

[26] Nesina IP, Iurchenko NP, Nespryadko SV (2014). The study of chromosomal instability in patients with endometrial cancer. Exp Oncol.

[27] Ferraro F, Gao F, Stockerl-Goldstein K (2018). Secondary acute lymphoblastic leukemia, a retrospective analysis from Washington University and meta-analysis of published data. Leuk Res.

[28] Giri S, Chi M, Johnson B (2015). Secondary acute lymphoblastic leukemia is an independent predictor of poor prognosis. Leuk Res.

[29] Aldoss I, Stiller T, Song J (2017). Philadelphia chromosome as a recurrent event among therapy related leukemia. Am J Hematol.

[30] Abou Dalle J, Jabbour E, Short NJ (2019). Treatment of Philadelphia chromosome positive acute lymphoblastic leukemia. Curr Treat Options Oncol.

